# Advances in Mitochondrial Dysfunction and Its Role in Cardiovascular Diseases

**DOI:** 10.3390/cells14201621

**Published:** 2025-10-17

**Authors:** Yan Qiu, Shuo Chang, Ye Zeng, Xiaoqi Wang

**Affiliations:** 1 Department of Cardiovascular Surgery, Fuwai Yunnan Hospital, Chinese Academy of Medical Sciences, Affiliated Cardiovascular Hospital of Kunming Medical University, Kunming 650102, China; qywvin@126.com (Y.Q.); cschl@126.com (S.C.); 2 Department of Cardiovascular Surgery, Fuwai Hospital, National Center for Cardiovascular Diseases, Chinese Academy of Medical Sciences and Peking Union Medical College, Beijing 100010, China; 3 Institute of Biomedical Engineering, West China School of Basic Medical Sciences and Forensic Medicine, Sichuan University, Chengdu 610041, China

**Keywords:** cardiovascular disease, mitochondrial dysfunction, oxidative stress, mitophagy, mitochondria dynamics, targeted therapy

## Abstract

Cardiovascular diseases (CVDs) remain the leading cause of morbidity and mortality worldwide and is attributed to complex pathophysiological mechanisms that surpass the traditional risk factors. Emerging evidence indicates that mitochondrial dysfunction plays a central role in CVD progression, linking impaired bioenergetics, oxidative stress imbalance, and defective mitochondrial quality control to endothelial dysfunction, myocardial injury, and adverse cardiac remodeling. However, the mechanistic interplay between mitochondrial dysfunction and CVD pathogenesis remains unclear. This review provides a comprehensive synthesis of recent knowledge, focusing on the dysregulation of mitochondrial energy metabolism, alterations in mitochondrial membrane potential, and disruptions in mitochondrial dynamics, including the balance of fusion and fission, mitophagy, and biogenesis. Furthermore, we critically evaluated emerging mitochondria-targeted therapeutic strategies, including pharmacological agents, gene therapies, and regenerative approaches. By bridging fundamental mitochondrial biology with clinical cardiology, this review underscores the critical translational challenges and opportunities in developing mitochondria-focused interventions. A deeper understanding of the mitochondrial mechanisms in CVD pathophysiology will offer novel diagnostic biomarkers and precision-targeted therapeutics, thereby transforming CVD management.

## 1. Introduction

Cardiovascular diseases (CVDs) are the leading cause of mortality worldwide [1]. Coronary heart disease (CHD) constitutes 16% of all deaths [2]. Despite advances in preventive and interventional cardiology, it imposes a significant clinical and socioeconomic burden [3]. The 2023 Annual Report on Cardiovascular Health and Disease in China indicates a continued rise in the incidence of CVD and related deaths in the Chinese population. Although lipid-lowering therapies and plaque stabilization have improved [4,5], these interventions do not fully address the cardiomyocyte metabolic dysfunction that drives disease progression [6]. Numerous traditional risk factors such as hypertension, dyslipidemia, and diabetes contribute to the pathogenesis of CVDs. However, accumulating evidence has highlighted mitochondrial dysfunction as a fundamental driver of disease progression, which remains unexplored [7]. Mitochondria are central to cardiac metabolism, producing over 90% of myocardial adenosine triphosphate (ATP) and tightly regulating the production of reactive oxygen species (ROS), calcium homeostasis, and apoptotic signaling [8]. Disruptions in these processes can trigger endothelial dysfunction, inflammatory cascades, and cardiomyocyte injury, which are hallmarks of CVDs [9].

Recent studies have revealed that mitochondrial impairment extends beyond energy deficiency to induce oxidative stress imbalance, aberrant mitochondrial membrane potential (MMP), defective fusion-fission dynamics, and impaired mitophagy [10,11]. For instance, mitochondrial dysfunction in heart failure (HF) compromises ATP production, leading to impaired cardiac contractility and diastolic dysfunction [12]. Similarly, mitochondrial dysfunction aggravates ischemia–reperfusion injury (IRI) in ischemic heart disease, causing cardiomyocyte apoptosis and irreversible tissue damage [13]. Dysregulated mitophagy exacerbates cellular stress, ROS accumulation, and apoptotic signaling. These dysfunctions collectively exacerbate ischemic injury, promote maladaptive cardiac remodeling, and accelerate atherosclerosis (AS) [14]. However, the mechanistic interplay between mitochondrial dysfunction and CVD pathogenesis remains unclear. Moreover, mitochondria-targeted therapies, including antioxidants, metabolic modulators, and gene-based interventions, demonstrate promise; however, their clinical translation is impeded by challenges, such as therapeutic specificity, delivery mechanisms, and long-term efficacy [15]. Lopaschuk et al. demonstrated that sodium-glucose cotransporter-2 (SGLT2) inhibitors (for instance, empagliflozin) provide cardioprotection through indirect mitochondrial modulation [16]. However, direct mitochondria-targeting agents are limited by their poor specificity [17]. Gammage et al. demonstrated that mitochondrial gene-editing tools are effective in vitro. However, their in vivo application is hindered by the dual barriers of cellular uptake and mitochondrial membrane penetration [18].

This review provides emerging evidence on the mechanistic associations between mitochondrial dysfunction and CVDs, focusing on the key molecular pathways that drive disease progression. We also critically evaluated the latest therapeutic strategies designed to restore mitochondrial homeostasis, emphasizing the translational challenges and future research directions. By integrating mitochondrial biology with cardiovascular medicine, we aimed to bridge the gap between mechanistic understanding and clinical application, thereby paving the way for precision-targeted therapies that could revolutionize the management of CVDs.

## 2. Mechanisms of Mitochondrial Dysfunction in CVDs

### 2.1. Mitochondrial ROS (mtROS) Generation and Amplification

Under physiological conditions, mtROS serve as signaling molecules that modulate adaptation to hypoxia and autophagy. However, ischemic insult or metabolic stress destabilizes the Electron Transport Chain (ETC), exacerbating electron leakage and converting mtROS into cytotoxic mediators [19]. This “ROS-induced ROS release” phenomenon spread oxidative stress to adjacent mitochondria and cardiomyocytes. This establishes a feedforward loop that drives the opening of the mitochondrial permeability transition pore (mPTP) and apoptosis [20]. Notably, mtROS synergizes with extramitochondrial oxidants to amplify lipid peroxidation and loss of membrane integrity, particularly in atherosclerotic plaques [21].

Mitochondrial DNA (mtDNA) is more susceptible to oxidative stress than nuclear DNA because of its lack of protective histones and inefficient DNA repair pathways [22]. A large-scale prospective cohort study of 21,870 individuals demonstrated that decreased mtDNA copy number (mtDNA-CN) independently predicted an increased incidence of cardiovascular diseases [23]. Furthermore, ROS-mediated mtDNA mutations can exacerbate mitochondrial dysfunction, resulting in a self-perpetuating cycle of oxidative stress and genetic instability [24]. Mechanistically, mtDNA acts as a damage-associated molecular pattern (DAMP). It promotes inflammation through numerous pathways, including cyclo-GMP-AMP synthase (cGAS)/interferon gene-stimulating factor (STING) signaling, inflammasome activation, and Toll-like receptor 9 signaling [25]. These inflammatory responses contribute to endothelial dysfunction and plaque formation, which are the key processes in AS. Moreover, mtDNA damage reduces ATP synthesis and MMP, a critical indicator of mitochondrial health. Decreased MMP increases the opening of the mPTP, an important regulator of cell death, thereby promoting apoptosis [26]. Since mitochondrial function depends on proteins encoded by both nuclear and mitochondrial DNA, mtDNA is considered a potential preclinical marker of AS and a therapeutic target [27]. For instance, mutations in the gene encoding the mitochondrial complex I ND1 subunit (A3397G) were detected in the heart tissue or serum of patients undergoing coronary artery bypass grafting (CABG), potentially linking mitochondrial dysfunction to the disease [28].

mtDNA heteroplasmy (for instance, A11467G, 576insC, and A1811G) in monocytes of obese and CHD patients has been associated with cardiovascular risk factors such as lipids, Body Mass Index, and carotid intima-media thickness [29]. Furthermore, increased mtDNA-CN in monocytes from CHD and obese patients is associated with increased secretion of the pro-inflammatory cytokine tumor necrosis factor-alpha (TNF-α), indicating a link between mitochondrial dysfunction and inflammation [30]. Targeting mtDNA repair mechanisms or reducing mtROS production may offer novel therapeutic strategies for CHD. Numerous studies have reported that targeting ROS generation or upstream pathways provides cardioprotection. For instance, myeloperoxidase (MPO) expression in endothelial colony-forming cells (ECFCs) is associated with increased mitochondrial dysfunction and ROS production in patients with CHD [31]. Endothelial S1PR2 induces excessive mitochondrial fission and ROS production through the RHO/ROCK1/DRP1 pathway, thereby exacerbating cardiac IRI [32]. Drugs such as artesunate can alleviate HF by inhibiting ROS production and improving mitochondrial damage via the SIRT1/FOXO3a/MnSOD pathway [33]. Perfluorooctane sulfonate and perfluorooctane sulfonamide induce oxidative stress-mediated cardiac defects via the peroxisome proliferator-activated receptor gamma (PPARγ) and aryl hydrocarbon receptor pathways, respectively [34]. Elevated oxidative stress markers, including iNOS, NOX2, nitrotyrosine, and 8-OHdG, have also been observed in septic cardiomyopathy [35]. Metals in PM2.5 can induce oxidative stress and myocardial IRI through mitochondrial accumulation [36]. Olive oil may ameliorate cardiac aging by reducing oxidative stress and modulating mitophagy and apoptosis-related genes [37].

### 2.2. Metabolic Dysfunction and Energetic Crisis

Cardiomyocytes exhibit unparalleled metabolic flexibility, relying primarily on mitochondrial oxidative phosphorylation (OXPHOS) for approximately 90% of their ATP, derived from a combination of coordinated fatty acid (FA) β-oxidation (FAO; 60–80%) and glucose oxidation (20–40%). The remainder is supplied by other metabolic pathways. However, the efficiency of energy production is reduced in pathological conditions such as HF, leading to a reduction in mitochondrial function, which affects the contractility and overall function of the heart [38]. Mitochondrial dysfunction is often triggered by an inadequate energy supply to endothelial cells. ATP deficiency impairs cardiomyocyte contraction because cardiac muscle contraction requires ATP to drive calcium ion transport and actin sliding. Studies have demonstrated that ATP depletion and acidosis can impair myocardial contractility and damage cell membrane pumps and ion channels [39]. These changes impair the ability of the heart to pump blood efficiently, thereby contributing to HF. The heart undergoes metabolic remodeling owing to energy impairment, which involves a shift in the energy substrate from FAs to glucose. Although this adaptation may help maintain cardiac function in the short term, it reduces ATP efficiency and exacerbates cardiac dysfunction over time [40]. This metabolic shift further aggravates the energy deficit, resulting in a vicious cycle that worsens HF.

Prolonged ATP underproduction leads to sustained deterioration of cardiac function and development of chronic HF (CHF). ATP deficiency plays a central role in these mechanisms by affecting cardiac metabolism, function, and structure. Mitochondrial dysfunction reduces ATP production and increases oxidative stress, thereby further damaging cardiomyocytes through lipid peroxidation, protein oxidation, and DNA damage [41]. These changes disrupt the intracellular environment and ultimately impair the overall function of the heart. In diabetic ischemic heart disease, mitochondrial ribosomal protein L7/L12 (MRPL12) levels are elevated, and its overexpression impairs MMP and respiratory capacity, implying a potential compensatory role in the pathophysiology of diabetic myocardial infarction (MI) [42]. In a model of repetitive myocardial stunning caused by chronic multi-vessel coronary stenosis, reduced FAO and enhanced metabolism of ketogenic amino acids were observed along with alterations in mitochondrial membrane phospholipid composition. These changes are consistent with impaired mitochondrial function and are associated with reduced nitric oxide (NO) and PPAR signaling pathways and decreased adenosine 5′-monophosphate-activated protein kinase (AMPK) activity [43]. Therapeutic interventions, such as 19,20-epoxydocosapentaenoic acid (19,20-EDP), can improve cardiac function and glucose oxidation rates after ischemic injury by directly activating mitochondrial SIRT3 [44]. Salidroside may mitigate myocardial IRI by activating Nuclear Factor Erythroid 2-Related Factor 2 (Nrf2) and modulating the AMPK/PGC-1α/PPARα pathway, thereby restoring mitochondrial homeostasis and improving ATP levels [45]. Parental obesity can exacerbate cardiac dysfunction after MI by affecting cardiac mitochondrial respiration and superoxide production in the offspring [46].

### 2.3. Calcium Homeostasis

Calcium ions (Ca^2+^) serve as key regulators of the mitochondrial redox balance and ATP synthesis. Mitochondria function as primary intracellular calcium storage compartments [47] and are essential for regulating multiple metabolic pathways, including lipid catabolism and Ca^2+^-dependent signal transduction [48]. In cardiac myocytes, Ca^2+^ shuttling between the endoplasmic reticulum (ER), cytosol, and mitochondrial matrix dynamically modulates OXPHOS, thereby sustaining the high-energy demands of myocardial contraction [49]. Ca^2+^ plays a central role in the regulation of excitation-contraction coupling, a crucial process for maintaining cardiac function. Under normal conditions, a slight increase in Ca^2+^ levels stimulates myocardial contraction, whereas mitochondria accumulate Ca^2+^ to support OXPHOS and ATP synthesis in the ETC. However, excessive intracellular Ca^2+^ accumulation disrupts mitochondrial integrity, leading to bioenergetic dysfunction, dysregulated cellular homeostasis, and activation of apoptosis through mitochondria-dependent pathways. Furthermore, higher Ca^2+^ levels exacerbate inflammatory responses by promoting the release of pro-inflammatory mediators. Imbalanced mitochondrial Ca^2+^ homeostasis inhibits Ca^2+^ reuptake in the sarcoplasmic reticulum (SR). This increases the Ca^2+^ efflux through the ryanodine receptor (RYR), leading to a temporary decrease in excitatory site activation. However, cytoplasmic Ca^2+^ initially increases, resulting in a Ca^2+^ overload [50]. Ca^2+^ overload can lead to the opening of the mPTP, increased mitochondrial oxidative stress, collapse of the MMP, disturbed ATP production, and necrosis of cardiomyocytes [51,52]. The mitochondrial calcium uniporter (MCU) complex, an ion channel situated in the inner mitochondrial membrane (IMM), is essential for preventing mitochondrial Ca^2+^ overload and maintaining Ca^2+^ homeostasis [53]. Moreover, H^+^/Ca^2+^ exchangers, such as the leucine zipper EF-containing transmembrane protein 1 (LETM1), regulate mitochondrial Ca^2+^ release in mammalian cells [54]. LETM1-deficient mice exhibit lower cytosolic Ca^2+^ levels, reduced mitochondrial Ca^2+^ uptake, and impaired glucose metabolism, highlighting the significance of Ca^2+^ regulation in cellular functions [55].

Ca^2+^ imbalance may exacerbate cardiovascular diseases. A prospective cohort study reported that Ca^2+^ supplementation, particularly in combination with vitamin D, increases the risk of coronary heart disease in individuals aged ≥ 52 years [56]. This increased risk may be attributed to vascular calcification, a process characterized by excessive Ca^2+^ deposition in arterial walls [57]. Paradoxically, low serum Ca^2+^ levels are considered an independent predictor of mortality in patients with coronary artery disease (CAD), highlighting the importance of maintaining optimum Ca^2+^ levels [58]. Ivabradine can mitigate doxorubicin-induced cardiotoxicity by improving mitochondrial function and restoring Ca^2+^ homeostasis [59]. Following MI, increased cellular-Src kinase activity leads to MCU tyrosine phosphorylation, thereby enhancing mitochondrial Ca^2+^ uptake, which in turn prolongs the QT interval and increases the risk of arrhythmias [60]. Similarly, MI-induced HF is characterized by an increased calpain-2-mediated cleavage of junctophilin-2 (JPH2). Targeted inhibition of this cleavage can improve SR Ca^2+^ handling and cardiac function [61]. Microvascular dysfunction after heart transplantation, although associated with increased mitochondrial density, may not impair excitation-contraction coupling (Ca^2+^ transients), potentially because of compensatory mechanisms [62].

### 2.4. Mitochondrial Quality Control (MQC)

**Figure 1:**
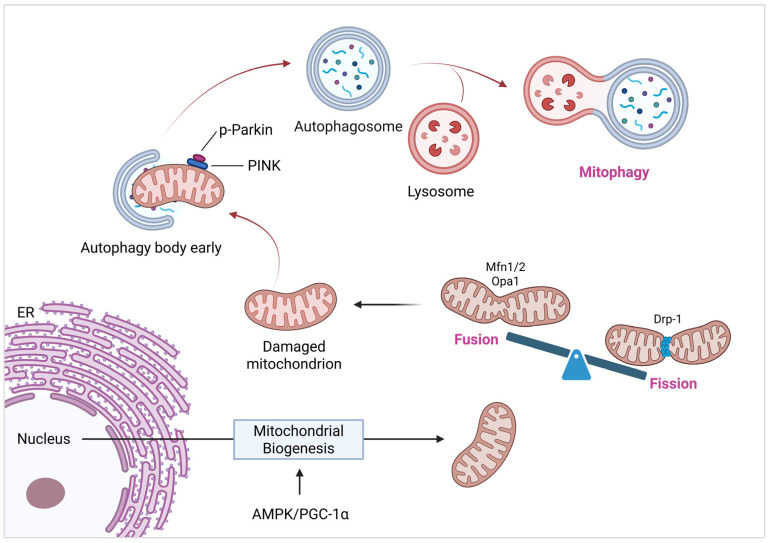
Mitochondrial quality control through dynamics and mitophagy. Mitochondrial integrity is maintained through coordinated fission and fusion, as well as mitophagy. Damaged fragments are segregated via Drp1-mediated fission and removed by autophagy. Mitochondrial fusion mediated by Mfn1/2 and OPA1 supports functional complementation. PGC-1α regulates mitochondrial biogenesis. Collectively, these processes maintain mitochondrial homeostasis. Abbreviations: AMPK, Adenosine 5′-monophosphate-activated Protein Kinase;Drp1, Dynamin-Related Protein 1; ER, endoplasmic reticulum; Mfn1/2, Mitofusins 1/2; OPA1, Optic Atrophy 1; PGC-1α, Peroxisome Proliferator-Activated Receptor-γ Coactivator-1α; PINK, PTEN-Induced Kinase.

MQC is a sophisticated regulatory network that involves mitochondrial biogenesis, dynamics (fusion and fission), and selective autophagy (mitophagy). These coordinated processes maintain the structure, population, and function of the mitochondria in cardiomyocytes (Figure 1). MQC disruption triggers mitochondrial dysfunction, leading to metabolic imbalances, Ca^2+^ dyshomeostasis, increased ROS production, and apoptotic activation. These events collectively exacerbate cardiovascular pathogenesis. Cardiac mitochondrial biogenesis increases mitochondrial quantity, facilitates mtDNA replication and repair, and upregulates mitochondrial protein synthesis [63]. Mitochondrial biogenesis expands the mitochondrial pool through the coordinated activation of PPAR-γ coactivator-1α (PGC-1α) and mitochondrial transcription factor A (TFAM), which upregulates nuclear respiratory factors (NRF1/2) and estrogen-related receptor (ERR)-dependent transcriptional programs [64]. This process is modulated by energy-sensing pathways, including AMPK (via PGC-1α phosphorylation at Thr177/Ser538) [65] and SIRT1/3 (through deacetylation of PGC-1α and metabolic regulators) [66], with therapeutic potential demonstrated by nicotinamide mononucleotide (NMN)-mediated SIRT3 restoration via AMPK/PGC-1α signaling [67]. Although PGC-1α overexpression suppresses excessive mitophagy via SIRT2 in annulus fibrosus cells [68], it also establishes a core regulatory network for mitochondrial biosynthesis. This occurs through the activation of transcription factors such as ERRs and NRF1/2, in conjunction with AMPK/SIRT signaling. For instance, Jiawei Dachaihu Tang protected mitochondrial function in AS mice with chronic unpredictable mild stress (CUMS) via the SIRT1/PGC-1α/TFAM/LON signaling pathway [69].

In addition to mitochondrial biosynthesis, the dynamic equilibrium of mitochondrial morphology (fusion and fission) is a central aspect of MQC [70]. Mitochondrial morphology is dynamically regulated by fusion mediated by mitofusins1/2 (Mfn1/2) and optic atrophy 1 (OPA1), whereas fission is dependent on dynamin-associated protein 1 (Drp1)/dynamin 2 (DNM2) [71,72]. Fusion promotes functional complementarity between mitochondria, whereas fission facilitates the segregation of damaged organelles, a balance that is disrupted in HF, as demonstrated by OPA1 deficiency-induced mtDNA depletion [73]. JNK-induced Mfn2 phosphorylation promotes fission [74], whereas S-nitrosylated Parkin inhibits Mfn1 degradation, thereby elongating mitochondria [75]. The dynamic balance between mitochondrial fusion, which contributes to functional complementarity and energy partitioning, and division, which promotes the isolation and clearance of damaged mitochondria, is crucial for maintaining cardiomyocyte homeostasis. Numerous studies have confirmed the role of imbalanced mitochondrial dynamics in the development of cardiac disease. For instance, echinacoside ameliorates ox-LDL-induced coronary artery endothelial cell dysfunction by regulating the mitochondrial fusion-fission balance through the activation of the Nrf2/PPARγ signaling pathway [76]. The mitochondrial fusion promoter M1 and fission inhibitor Mdivi-1 can attenuate the mitochondrial dynamic imbalance and mitigate cardiac remodeling and dysfunction after MI in rats [77]. In the myocardium of broilers with pulmonary hypertension (PH), the expression of fusion-related proteins decreases, whereas fission-related proteins increase (Drp1 and Mff) [78]. Copper deficiency reduces the expression of mitochondrial fusion proteins and increases the expression of fission proteins, thereby causing cardiac injury [79]. QiShenYiQi Pills (QSYQ) can ameliorate ischemic HF by downregulating MCU, MARCHF5, and MTFP1, thereby inhibiting Drp1-induced excessive mitochondrial fission [80]. Longxuetongluo Capsule (LTC) regulates mitochondrial morphology by increasing Mfn2 expression and decreasing p-Drp1 levels, thereby mitigating myocardial IRI [81]. Mitoquinone (MitoQ) combined with alpha-lipoic acid improves mitochondrial dynamics in elderly rat with myocardial IRI by upregulating Mfn1/Mfn2 and downregulating Drp1/Fis1 [82]. The lncRNA Oip5-as1 inhibits excessive mitochondrial fission in myocardial IRI by regulating the AKAP1/CaN/Drp1 pathway and inhibiting Drp1 Ser637 dephosphorylation [83]. Jin-Xin-Kang (JXK) improves mitochondrial function and treats CHF by inhibiting the CaN/Drp1 pathway [84].

Mitophagy, a selective autophagy pathway essential for eliminating depolarized or damaged mitochondria, is regulated by context-dependent molecular switches. Selective mitophagy eliminates dysfunctional mitochondria via the PTEN-induced kinase 1 (PINK1)/Parkin pathway and other pathways [85]. Quercetin enhances PINK1/Parkin activity through SIRT5-dependent DNA-PKcs stabilization, suppressing mixed lineage kinase domain-like protein (MLKL)-mediated necroptosis [86]. Conversely, calcineurin overexpression induces mPTP-dependent Parkin activation [87]. The antagonism of necroptosis-dependent PINK1-mediated mitophagy is also dependent on phosphoglycerate mutase family member 5 (PGAM5) [88]. The therapeutic potential for cardiovascular diseases can be achieved by restoring mitochondrial homeostasis and inhibiting pathological cell death by targeting MQC components, such as AMPK/PGC-1α, Drp1, and PINK1. For instance, Puerarin may alleviate ER stress and mitochondrial dysfunction in myocardial ischemic injury by upregulating the KLF4/Mzb1 pathway, potentially via mitophagy regulation [89]. Prosapogenin (a GAS6 receptor agonist) regulates mitophagy and inhibits Lipopolysaccharide-induced cardiomyocyte necroptosis by targeting the PGAM5-voltage-dependent anion channel 1 (VDAC1) axis [90]. Ivabradine mitigates doxorubicin-induced cardiotoxicity by improving mitochondrial function, including dynamics and autophagy [59]. QSYQ alleviates ischemia-induced HF by inhibiting MCU/MARCHF5/MTFP1-Drp1-induced mitochondrial fission, which is closely associated with mitophagy [80]. In a study utilizing a rat model of chemically induced aging via D-galactose (D-GAL), olive oil may improve cardiac aging by enhancing the genes that mediate mitophagy. However, these findings are primarily based on a specific experimental aging model, and direct extrapolation to human coronary heart disease prevention requires caution and further validation through clinical studies [37]. Humanin inhibits lymphatic endothelial cell dysfunction and alleviates myocardial IRI via BNIP3-mediated mitophagy [91]. Mir221/Mir222-enriched adipose stem cell-derived exosomes regulate mitophagy and apoptosis by targeting the BNIP3-MAP1LC3B-BBC3/PUMA pathway, mitigating PM2.5-exacerbated myocardial IRI [92]. Po-Ge-Jiu-Xin decoction (PGJXD) may alleviate sepsis-induced cardiomyopathy by modulating PINK1/Parkin-mediated mitophagy [93]. Xinyang tablet (XYT) alleviated cardiac dysfunction in a pressure overload model by regulating the RIPK3/FUNDC1-mediated mitochondrial unfolded protein response and mitophagy [94].

## 3. Mitochondrial Dysfunction and Specific Cardiac Conditions

### 3.1. Atherosclerosis

#### 3.1.1. Mitochondrial Damage and Endothelial Dysfunction

**Figure 2:**
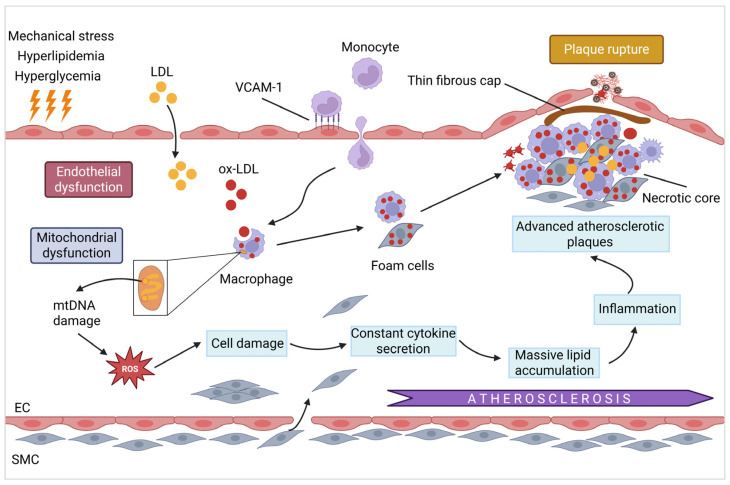
Mitochondrial dysfunction in atherosclerosis. Foam cell formation is stimulated by ox-LDL-induced endothelial activation and monocyte recruitment. Mitochondrial dysfunction in macrophages increases ROS production and inflammatory signaling, disrupts lipid metabolism, and accelerates necrotic core formation, thereby exacerbating plaque progression. Abbreviations: EC, endothelial cells; LDL, low-density lipoprotein; SMC, smooth muscle cells; VCAM-1, vascular cell adhesion molecule 1.

Atherosclerosis (AS) is caused by endothelial injury and mitochondrial dysfunction. Injury to vascular endothelial cells is a pivotal initiating factor, and mitochondrial dysfunction plays a key role (Figure 2). Damaged mitochondria release the pro-inflammatory cytokines interleukin-6 (IL-6) and TNF-α, recruiting immune cells to the vascular wall and accelerating plaque formation [95]. Furthermore, mtDNA-derived DAMPs exacerbate chronic inflammation and immune activation, thereby accelerating the progression of AS [96]. Endothelial dysfunction, a hallmark of early AS, is characterized by increased permeability and inflammatory cell recruitment [97]. Injured endothelial cells allow low-density lipoprotein (LDL) to accumulate in the subendothelial space. Oxidized LDL (ox-LDL) upregulates the adhesion molecules ICAM-1 and VCAM-1, perpetuating endothelial damage and promoting monocyte adhesion [98]. These monocytes differentiate into macrophages, engulf ox-LDL, and transform into foam cells, thereby producing fatty streaks, which are the earliest AS lesions [99]. Several studies have highlighted the role of endothelial mitochondrial dysfunction in AS. For instance, EP300 ameliorates endothelial injury and mitochondrial dysfunction in CHD by regulating histone acetylation of the Suppressor of Cytokine Signaling 1 (SOCS1) promoter [100]. Echinacoside improves ox-LDL-induced coronary artery endothelial cell dysfunction by activating the Nrf2/PPARγ signaling pathway [76]. MPO expression in ECFCs is associated with mitochondrial dysfunction in patients with CAD [31]. Endothelial S1PR2 exacerbates cardiac IRI by inducing mitochondrial fission and ROS production, indicating its role in vascular injury [32]. In the human microvascular endothelium, ceramide produced by neutral sphingomyelinase (NSmase) is essential for maintaining NO signaling; however, in patients with CAD, downstream signaling is disrupted, leading to hydrogen peroxide (H_2_O_2_) production over NO [101]. Fluctuations in lipid levels can induce endothelial dysfunction by increasing inflammation and oxidative stress [102]. The lncRNA NORAD promotes endothelial cell proliferation and prevents ferroptosis by regulating the miR-106a/CCND1 axis [103]. In type 2 diabetes, higher levels of extracellular nicotinamide phosphoribosyltransferase (eNAMPT) promote coronary microvascular disease (CMD) via TLR4, and inhibiting eNAMPT improves endothelial function [104].

#### 3.1.2. Mitochondrial Dysfunction and Plaque Instability/Rupture

Mitochondrial damage contributes to plaque destabilization by changing the cellular composition (reduced smooth muscle cells (SMCs) and increased macrophages) and weakening the fibrous cap [105,106,107]. As plaques develop, SMCs migrate from the media to the intima, where they proliferate and secrete extracellular matrix (ECM), forming a fibrous cap over the lipid core [108]. However, the instability of advanced plaques may be increased by hemorrhage, necrosis, and calcification. Unstable plaques are prone to rupture, leading to thrombosis and acute cardiovascular events [109]. Mitochondrial dysfunction exacerbates atherosclerotic plaque instability by amplifying oxidative stress and inflammation. Excessive ROS production in endothelial cells and macrophages upregulates adhesion molecules and pro-inflammatory cytokines, leading to leukocyte infiltration and expansion of the necrotic core [110]. In vascular SMCs (VSMCs), the integrity of the fibrous cap is collectively weakened by mitochondrial defects, which impair collagen synthesis while activating matrix metalloproteinases [110]. Clinical evidence from intravascular ultrasound studies confirms that mitochondrial DNA damage is associated with vulnerable plaque features, such as thin caps and large lipid cores [111]. Furthermore, aldehyde dehydrogenase 2 (ALDH2) deficiency [112] and age-related hyperlipidemia [113] accelerate ROS-mediated VSMC senescence via p53/p21 activation. Notably, the pharmacological targeting of mitochondrial ROS or downstream effectors has emerged as a promising strategy to stabilize plaques and prevent acute cardiovascular events. Monocyte mtDNA heteroplasmy [29] and increased mtDNA-CN associated with TNF-α secretion [30] further link mitochondrial damage to AS inflammation. CUMS can promote AS progression by exacerbating mitochondrial dysfunction, whereas traditional Chinese medicine formulas, such as Jiawei Dachaihu Tang, may mitigate AS and stress by improving mitochondrial function through regulation of the SIRT1/PGC-1α pathway [69]. Vascular aging is also closely associated with AS and involves numerous mechanisms including oxidative stress, inflammation, endothelial dysfunction, and vascular cell senescence [114].

#### 3.1.3. Potential Therapeutic Targets

Mitochondrial dysfunction is a well-established cause of inflammation, primarily through increased mitochondrial ROS production and oxidative stress. When mitochondria are damaged, they release mtDNA, which acts as a DAMP and induces robust immune responses [115]. This DAMP recognition initiates NOD-Like Receptor Protein 3 (NLRP3) inflammasome assembly, resulting in the activation of potent pro-inflammatory signaling cascades [116]. The transcriptional coactivator PGC-1α plays an important regulatory role in this process by upregulating antioxidant genes and reducing ROS levels, thereby promoting alternative macrophage activation. Notably, PGC-1α overexpression increases mitochondrial biogenesis while suppressing the production of pro-inflammatory cytokines [117] and has been demonstrated to inhibit atherosclerotic lesion formation [118]. Autophagy is another critical regulatory mechanism that effectively suppresses NLRP3 inflammasome activation [119] and protects VSMCs from apoptosis induced by either pro-atherosclerotic LDL modifications [120] or autophagy deficiency [121]. These findings highlight the therapeutic potential of targeting mitochondrial inflammatory pathways. In line with this concept, colchicine—a potent anti-inflammatory agent—has shown promise in reducing cardiovascular events in large-scale trials such as COLCOT and LoDoCo2, although a clear mortality benefit has not been established. This provides cautious optimism for anti-inflammatory strategies in this patient population [122,123].

### 3.2. Ischemia–Reperfusion Injury (IRI)

#### 3.2.1. Oxidative Stress and mPTP Activation

**Figure 3:**
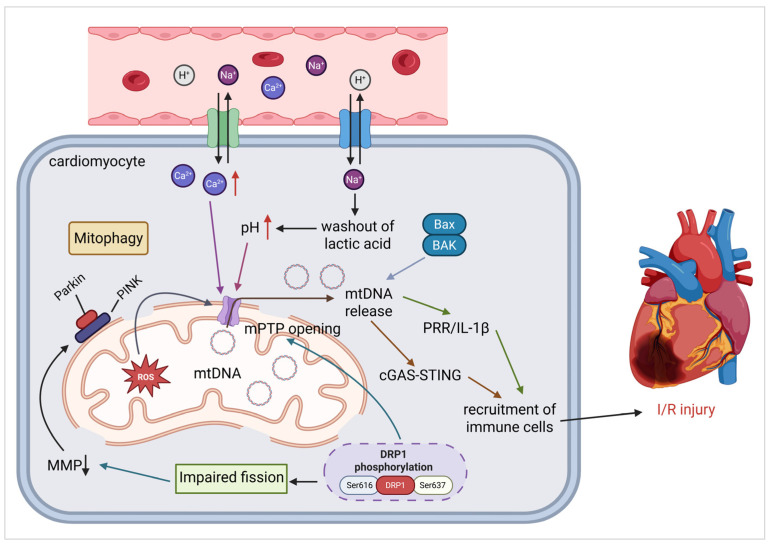
Mitochondrial dysfunction in myocardial IRI. Reperfusion triggers an intracellular Na^+^/Ca^2+^ overload and excessive mitochondrial ROS production. The opening of the mPTP exacerbates damage, facilitating further ROS release and immune cell infiltration via DAMPs, thereby amplifying IRI. Abbreviations: cGAS, cyclic GMP–AMP syntheses; MMP, mitochondrial membrane potential; mPTP, mitochondrial permeability transition pore; PRR, pattern recognition receptor; STING, stimulator of interferon genes.

Ischemic heart disease remains the leading cause of global mortality, with acute myocardial ischemia being the most common clinical manifestation. Timely reperfusion is critical for salvaging ischemic myocardium. While reperfusion is lifesaving, it paradoxically exacerbates injury via mitochondrial dysfunction, a process induced by ROS, Ca^2+^ overload, and mPTP opening (Figure 3).

Under hypoxic conditions, cells undergo a metabolic shift toward anaerobic glycolysis, leading to the accumulation of lactic acid and subsequent intracellular acidification. This acidic environment activates the Na^+^/H^+^ exchanger (NHE) [124], whereas the Na^+^/K^+^-ATPase activity is impaired by concurrent ATP depletion, leading to intracellular Na^+^ accumulation. Upon reperfusion, oxygen restoration rapidly resumes ATP production, developing a steep transcellular Na^+^ gradient that further activates the NHE. The resulting Na^+^ influx drives excessive Ca^2+^ uptake via Na^+^/Ca^2+^ exchange, thereby initiating calcium overload. This process is exacerbated by I/R-induced catecholamine release, which activates G protein-coupled receptor signaling pathways. Phospholipase C activation promotes Ca^2+^ release from ER stores, further contributing to cytosolic Ca^2+^ overload [125]. The combined effects of ROS production and Ca^2+^ overload synergistically induce mPTP opening, triggering mitochondrial swelling, cytochrome c release, and ultimately, caspase-dependent apoptosis. The mPTP is a high-conductance, non-selective channel implicated primarily in oncotic necrosis and necroptosis [126]. Its component or associated proteins include the voltage-dependent anion channel (VDAC) and adenine nucleotide translocase (ANT), which interact to bridge the inner and outer mitochondrial membranes, along with cyclophilin-D (CypD), the benzodiazepine receptor (BDR), and hexokinase [127]. Notably, under conditions of oxidative stress, the mitochondrial F-ATP synthases can be turned into Ca^2+^-dependent channels whose electrophysiological properties match those of the corresponding mPTPs [128]. Under ischemia–reperfusion (I/R) conditions, increases in matrix Ca^2+^ levels and oxygen free radicals cause the mPTP to open, leading to a rapid loss of MMP and unregulated release of mitochondrial components [129]. Furthermore, the specific composition of a mitochondrial apoptosis channel (MAC) has not been fully determined, but it is known that the interactions of Bax, Bak, and tBID of the Bcl-2 family of proteins with the outer mitochondrial membrane control the mitochondrial outer membrane permeabilization (MOMP) [130]. Both mPTP and MAC are regulated by Bcl-2 proteins and can be opened by elevated levels of Ca^2+^ and oxidative stress [131]. Activation of the MAC leads to the release of the electron carrier cytochrome c, which triggers apoptosis by activating the apoptosome, which in turn activates caspases and DNAses [132]. Consequently, maintaining the integrity of mitochondrial ion channels and MMP is a primary focus for cardioprotective strategies [133]. These pathological changes collectively impair cellular membrane integrity and mitochondrial ATP synthesis, thereby creating a vicious cycle that exacerbates IRI [134].

#### 3.2.2. Mitochondrial Fission and Bioenergetic Failure

The second pathological mechanism of IRI involves dysregulated mitochondrial dynamics, including fission, fusion, and mitophagy. Under basal conditions, mitochondrial fission protein Drp1 remains cytosolic and inactive. During cellular stress, post-translational modifications, particularly phosphorylation, induce conformational changes in Drp1, facilitating its translocation to mitochondrial receptors and subsequent fission initiation. Ser616 and Ser637 are two critical phosphorylation sites in Drp1. Phosphorylation at Ser616 enhances Drp1-mitochondrial membrane binding and promotes fission [135], whereas Ser637 phosphorylation inhibits Drp1 oligomerization and suppresses fission [136]. During reperfusion, the increased activity of fission-promoting kinases and the suppressed activity of fission-inhibiting phosphatases shift the balance toward pro-fission phosphorylation. Particularly, Ser616 phosphorylation increases, whereas Ser637 phosphorylation decreases [137]. This transition induces pathological mitochondrial fragmentation, resulting in MMP collapse and mPTP opening. Consequently, ATP depletion and cytochrome c release trigger cardiomyocyte apoptosis [138]. Mitochondrial fusion, regulated by OPA1 and Mfn1/2, counteracts pathological fission and maintains mitochondrial homeostasis. However, during reperfusion, Ca^2+^ overload downregulates OPA1 and Mfn2 expression, impairing fusion and exacerbating the imbalance toward excessive fission. In addition to the dysregulation of fission and fusion, impaired mitophagy disrupts MQC. During I/R, MMP depolarization recruits PINK1 to the mitochondrial membranes, where it phosphorylates and activates Parkin, resulting in excessive mitophagy [139]. Conversely, FUNDC1-mediated mitophagy is suppressed by which phosphorylates FUNDC1 to inhibit its activity and block its response to MMP depolarization [140], resulting in premature clearance of functional mitochondria while failing to remove damaged mitochondria, further exacerbating cardiomyocyte apoptosis.

Numerous studies have supported the critical roles of mitochondrial dynamics and mitophagy in IRI. For instance, the mitochondrial fusion promoter M1 and fission inhibitor Mdivi-1 improve cardiac function and mitochondrial dynamic imbalance in rats after MI [77]. Endothelial S1PR2 induces excessive mitochondrial fission and ROS production via the RHO/ROCK1/Drp1 pathway, leading to NLRP3 inflammasome activation, pyroptosis, and IRI exacerbation [32]. LTC mitigates myocardial IRI by regulating Mfn2 and p-Drp1 expression to restore mitochondrial morphology [81]. By regulating mitochondrial dynamics-related gene expression, MitoQ combined with alpha-lipoic acid improves myocardial IRI in elderly rats [82]. The lncRNA Oip5-as1 mitigates myocardial IRI by inhibiting excessive mitochondrial fission via the AKAP1/CaN/DRP1 pathway [83]. Humanin inhibits lymphatic endothelial cell dysfunction and mitigates myocardial IRI through BNIP3-mediated mitophagy [91]. Mir221/Mir222-enriched ADSC exosomes alleviate PM2.5-exacerbated myocardial IRI by regulating mitophagy by targeting the BNIP3/MAP1LC3B/BBC3 pathway [92]. Salidroside improves myocardial IRI by activating Nrf2, which induces autophagy and mitophagy, thereby improving mitochondrial dynamics imbalance [45]. Mitochondria-rich sEVs derived from cardiac fibroblasts can modulate tissue inflammation and post-MI ventricular remodeling via the NLRP3 pathway [141]. In summary, reperfusion-induced dysregulation of mitochondrial fission, fusion, and mitophagy collectively disrupts mitochondrial homeostasis, resulting in MMP collapse, mPTP opening, and ultimately, cardiomyocyte death. Targeting these dynamics may represent a potential therapeutic strategy for IRI.

#### 3.2.3. mtDNA Mutant and Release

ROS are the primary drivers of mtDNA mutations, although replication errors and relatively inefficient mtDNA repair capacity compared to nuclear DNA also contribute. Accumulated mtDNA damage amplifies ROS production, creating a self-perpetuating, vicious cycle that aggravates mitochondrial dysfunction. Classically, mtDNA release has been attributed to the opening of the mPTP [142]. However, additional molecular pathways also govern mtDNA release under oxidative stress, including the following:

1) Oligomerization of voltage-dependent anion channels (VDACs). In response to oxidative stress, VDAC proteins oligomerize within the mitochondrial outer membrane, promoting mitochondrial outer membrane permeabilization (MOMP) and facilitating the release of mtDNA into the cytosol [143].

2) Bax/BAK-mediated pore expansion. Following MOMP, the pro-apoptotic Bcl-2 family proteins Bax and BAK enlarge the outer mitochondrial membrane (OMM) pores, leading to mitochondrial inner membrane permeabilization (MIMP) and extrusion of mtDNA [144].

Once released, mutant mtDNA can localize to either intracellular or extracellular compartments, activating distinct inflammatory signaling pathways.

(1) Intracellular mtDNA acts as a DAMP by engaging pattern recognition receptors (PRRs), which initiate sterile inflammation and promote the secretion of cytokines, such as IL-1β and IL-10.

(2) Cytosolic mtDNA stimulates the cGAS/STING pathway, inducing type I interferon (IFN) production and amplifying innate immune responses [145].

#### 3.2.4. Potential Therapeutic Strategies

mPTP is a critical regulator of IRI, and its inhibition is recognized as a key cardioprotective mechanism during pre- and post-ischemic conditioning [146]. Upon opening, mPTP induces mitochondrial matrix swelling and outer mitochondrial membrane (OMM) rupture, leading to the release of pro-apoptotic factors and mtDNA, which are major triggers of sterile inflammation. Given its central role in mitochondrial dysfunction, the mPTP has emerged as a promising therapeutic target for cardioprotection. Preclinical studies have demonstrated that pharmacological mPTP inhibitors, such as cyclosporine A, reduce infarct size by 30–40% in animal models [147]. Clinically, the modulation of mPTP activity may mitigate myocardial IRI. However, further validation using human trials is required. The fundamental cause of IRI is mitochondrial dysfunction, which connects oxidative stress, mPTP activation, and fission/fusion imbalance with cardiomyocyte death. Targeting these pathways has therapeutic potential, and mtDNA detection may refine risk stratification. Future studies should investigate the potential of combinatorial strategies to mitigate reperfusion injuries. Several potential therapeutic approaches that target these mechanisms have been investigated. L-theanine may alleviate oxidative stress and mitochondrial dysfunction in IRI by positively regulating antioxidant responses [148]. Puerarin can improve myocardial ischemic injury and ER stress by upregulating the Mzb1 signaling pathway [89]. 19,20-EDP exerts cardioprotective effects by directly activating mitochondrial SIRT3 [44]. MitoQ combined with alpha-lipoic acid exhibited protective effects against myocardial IRI in elderly rats [82]. *Humulus lupulus* (hops) extract can prevent cardiac mitochondrial and contractile ischemic dysfunction through the production of NO and the activation of mKATP channels [149]. Myricetin combined with exercise may mitigate MI in rats by inhibiting the Nrf2/HO-1 antioxidant pathway [150]. Salvianolic acid B counteracts myocardial IRI by inhibiting cardiomyocyte apoptosis by regulating Bax/Bcl-2/caspase-3 and JNK/p38 pathways [151]. The regulation of mitochondrial dynamics is associated with the inflammatory pathways in IRI. For instance, hydrogen sulfide has been demonstrated to protects against myocardial IRI by inhibiting NLRP3 inflammasome activation and regulating mitochondrial dynamics [152].

### 3.3. Heart Failure

#### 3.3.1. Metabolic Dysfunction and Oxidative Stress

**Figure 4:**
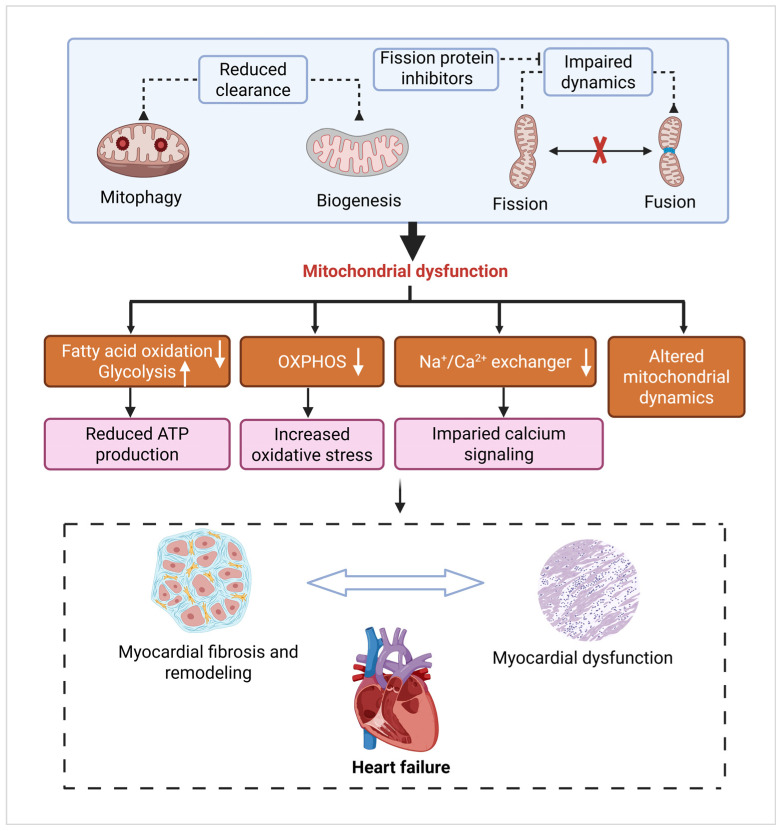
Mitochondrial dysfunction in HF. Dysfunctional mitochondria contribute to HF by impairing ATP production and elevating ROS levels, which initiate a cascade of cellular damage, inflammation, and progressive cardiac dysfunction. Abbreviations: ATP, adenosine triphosphate; OXPHOS, oxidative phosphorylation. Upward arrows indicate activation; downward arrows indicate inhibition.

HF is a complex clinical syndrome characterized by impaired ventricular filling or ejection owing to structural or functional cardiac abnormalities, a condition clinically stratified into three distinct subtypes based on left ventricular ejection fraction (LVEF): HF with preserved ejection fraction (HFpEF, LVEF ≥ 50%), HF with reduced ejection fraction (HFrEF, LVEF < 40%), and HF with mid-range ejection fraction (HFmrEF, LVEF 40–49%) [153,154]. While all HF phenotypes share a common denominator of bioenergetic insufficiency, the specific nature of mitochondrial impairment vary across these subtypes, driving disease progression through energy depletion, oxidative stress, and adverse cardiac remodeling [155]. In end-stage HF, the activities of Krebs cycle enzymes and oxidoreductases are significantly reduced [156]. Furthermore, mtDNA replication is severely impaired, resulting in the depletion of mtDNA-encoded proteins and disruption of mitochondrial biogenesis (Figure 4).

The heart requires a constant supply of ATP to sustain contraction, with metabolic flexibility switching between FAs and glucose, which is critical for adaptation. In healthy adults, approximately 70% of cardiac ATP is derived from FAO, fulfilling nearly 100% of the energy demand during fasting [157]. Alternative substrates such as glucose and lactate contribute minimally [158]. In HF, cardiomyocytes shift from FAO to glucose dependency, and this adaptation improves oxygen efficiency (glucose oxidation consumes less oxygen (O_2_) than FAO), which is accompanied by impaired OXPHOS and energy depletion [159]. Although glucose boosts ATP/O_2_ stoichiometry [160], its benefits are limited by the downregulation of mitochondrial pyruvate carrier (MPC) in HF [161]. Restoring MPC expression using cardiac assist devices [162] or genetic interventions rescues contractile dysfunction in mice, highlighting the role of pyruvate metabolism in cardiac energetics [163]. Future therapies may combine MPC modulation with metabolic substrates to optimize HF treatment. Recent studies indicate that mitochondrial dysfunction plays a pivotal role in the pathogenesis of HFpEF. Research demonstrates that mitochondrial function is markedly impaired in HFpEF patients, manifested by reduced respiratory capacity, diminished oxidative phosphorylation, and decreased levels of tricarboxylic acid cycle metabolites [164]. Moreover, mitochondrial hyperacetylation has been observed in cardiac tissue from HFpEF patients, closely associated with reduced NAD^+^/NADH ratios and impaired mitochondrial function [165,166]. This dysfunction not only disrupts cardiac energy metabolism but also diminishes peripheral skeletal muscle oxygen utilization, thereby precipitating exercise intolerance [167,168]. In contrast to HFpEF, HFrEF is primarily characterized by markedly reduced left ventricular systolic function, accompanied by myocardial cell apoptosis and fibrosis. Although the pathophysiological features of HFmEF lie between those of HFpEF and HFrEF, its manifestations of mitochondrial dysfunction are more similar to those seen in HFpEF. Studies indicate that in HFrEF patients, impaired cardiac mitochondrial function primarily manifests as increased oxidative stress and mitochondrial DNA damage, whereas HFmEF patients exhibit mitochondrial metabolic abnormalities similar to those seen in HFpEF [164]. In HFpEF, mitochondrial dysfunction directly impairs energy supply to cardiac and peripheral tissues, subsequently triggering diastolic dysfunction and exercise intolerance [167]. Furthermore, mitochondrial abnormalities exacerbate the pathological process of HFpEF by influencing inflammatory responses and oxidative stress [169].

#### 3.3.2. Fibrosis and Cardiac Remodeling

Furthermore, HF progression involves fibrosis and cardiac remodeling, mediated by angiotensin-II, norepinephrine, TNF-α, and mechanical stress, which activate protein kinase Cepsilon (PKC), mitogen-activated protein kinase (MAPK), phosphatidylinositol-3-hydroxykinase (PI3K), Jun N-terminal kinase (JNK), and nuclear factor-κB (NF-κB) pathways [170]. NF-κB activation upregulates MMPs, leading to ECM degradation and cardiomyocyte apoptosis, which accelerate ventricular remodeling [171]. For instance, in a porcine model of pacing-induced supraventricular tachycardia, MMP-1, MMP-2, and MMP-3 were demonstrated to induce left ventricular dysfunction and dilation within seven days [172].

#### 3.3.3. Mitochondrial Dynamics and Mitophagy Defects

Mitochondrial dysfunction in HF leads to impaired mitochondrial biogenesis. At the subcellular level, the integrity of the mitochondrial network is compromised in HF through a pronounced dysregulation of the dynamic processes of fusion and fission [173]. A hallmark of the failing myocardium is a shift in the balance of mitochondrial dynamics towards excessive fragmentation, driven by a downregulation of fusion mediators such as MFN2 and OPA1, coupled with an upregulation of the fission protein Drp1 [173,174]. This fragmented mitochondrial phenotype disrupts the efficient operation of the electron transport chain, ultimately culminating in deficient ATP synthesis [175,176]. The internal architecture of mitochondria, essential for optimal respiratory function, is maintained by the mitochondrial contact site and cristae organizing system (MICOS) complex [177]. Disruption of this complex and the consequent aberrations in cristae morphology are frequently observed in HF, impairing the supramolecular organization of respiratory complexes and exacerbating the bioenergetic crisis [178,179]. Studies in human and rat models of HF have revealed that mitochondria are small and fragmented, with low levels of OPA1. This suggests that mitochondrial fission is involved in cardiac remodeling [119]. Similarly, Ca^2+^ overload stimulates mitochondrial fission and fragmentation [120]. Furthermore, defective mitochondrial autophagy in HF impairs myocardial function because damaged and non-functional mitochondria are an important source of ROS [180]. For instance, in an experimental model of parkin-knockout Drosophila, it was observed that inhibition of mitochondrial autophagy increases the number of dysfunctional mitochondria in the cardiac tubules, thereby developing dilated cardiomyopathy [181]. Several studies have explored the roles of mitochondrial dynamics and mitophagy in HF. For instance, QSYQ mitigates ischemia-induced HF by inhibiting MCU/MARCHF5/MTFP1-DRP1-driven mitochondrial fission [80]. PGJXD may alleviate sepsis-induced cardiomyopathy by modulating PINK1/Parkin-mediated mitophagy [93]. XYT alleviates pressure overload-induced HF by regulating RIPK3/FUNDC1-mediated mitochondrial unfolded protein response and mitophagy [94]. Accordingly, further studies on HF mitochondrial function are crucial because of the central role of mitochondria in energy production.

#### 3.3.4. Potential Therapeutic Targets and Modulators

Given the central role of mitochondrial dysfunction in HF, therapeutic strategies have been designed to restore metabolic flexibility, enhance biogenesis, and improve quality control [182]. Effective therapies include modulators of FA metabolism, glucose metabolism, mitochondrial OXPHOS, antioxidants, and MQC [183]. L-carnitine restores ventricular carnitine levels and reduces fibrosis in Heart failure with preserved ejection fraction models [184], with clinical benefits in heart failure of ischemic origin [185]. In addition to its role in regulating glucose metabolism, SGLT2 inhibitors have been demonstrated to increase FAO and ketogenesis and rebalance the relationship between glycolysis and OXPHOS [186,187]. AMPK activation (for instance, metformin) and stimulation of the NO/sGC/cGMP pathway enhance biogenesis, thereby improving energy supply [164]. Coenzyme Q10 (CoQ10) supplementation reduces hypertrophy and fibrosis in diabetic cardiomyopathy by scavenging ROS [188]. MPC inhibitors may attenuate oxidative stress by modulating pyruvate-driven OXPHOS [189]. The integration of these approaches could synergistically rescue mitochondrial function and offer a multifaceted therapeutic strategy for HF treatment. Other potential therapies include the following: artesunate mitigating doxorubicin-induced HF via the SIRT1/FOXO3a/MnSOD pathway [33]; inhibition of p53 acetylation improving pressure overload-induced CMD and HFpEF [190]; Supplementation with nicotinamide riboside can restore the NAD^+^/NADH ratio and reduce the acetylation levels of mitochondrial proteins, thereby improving mitochondrial function and the HFpEF phenotype [191]. Furthermore, nitro-oleic acid significantly enhances cardiac function in HFpEF mice by activating the AMPK signaling pathway, thereby boosting mitochondrial respiratory capacity and metabolic function [192]. Buyang Huanwu decoction (BYHWD) improving cardiac function after MI by regulating the PI3K/Rap1/integrin α(IIb)β(3) pathway [193]; maternal ketone supplementation improving neonatal cardiac dysfunction caused by perinatal iron deficiency [194]; JXK improving mitochondrial function to treat CHF by inhibiting the CaN/Drp1 pathway [84]; Gualou Xiebai Banxia Decoction (GXBD) inhibiting post-MI cardiomyocyte apoptosis via activating ALDH2 [195]; Shikonin mitigating doxorubicin-induced cardiotoxicity via the Mst1/Nrf2 pathway [196]; combination or individual use of dapagliflozin and trimetazidine protecting against diabetic doxorubicin-induced cardiotoxicity by mitigating ER stress [197]. Interactions between dietary flavonoids and gut microbiota may also play a protective role in HF [198]. Exercise training is a cornerstone of heart failure management and a Class I recommendation in clinical guidelines [199,200]. A wide range of modalities, including aerobic, resistance, and high-intensity interval training, have been shown to improve exercise tolerance, quality of life, and clinical outcomes [201,202,203]. At a molecular level, exercise promotes beneficial cardiac remodeling and enhances mitochondrial function by stimulating biogenesis through the AMPK/PGC-1α pathway, improving respiratory efficiency, and reducing oxidative stress [204,205,206]. These adaptations collectively improve cardiac energetics and tolerance to ischemic stress.

## 4. Potential of Mitochondrial Dysfunction as a Biomarker and Therapeutic Target

### 4.1. Prospects of Mitochondria-Related Indicators in CVD Diagnosis

#### 4.1.1. GDF-15 and FGF-21

Recent advances in mitochondrial research have identified promising biomarkers for diagnosing and monitoring mitochondrial dysfunction. Growth differentiation factor 15 (GDF-15) and fibroblast growth factor 21 (FGF-21) have emerged as key candidates, although their diagnostic utility remains under investigation [207]. GDF-15 and FGF-21 expression increases during mitochondrial stress (for instance, ROS overproduction), rendering them surrogate markers of dysfunction [208,209]. FGF-21, primarily associated with muscle-expressed mitochondrial diseases, plays a dual role in metabolic regulation and mitochondrial health assessment, with elevated levels often suggesting dysfunction [210]. GDF-15, another critical biomarker, reflects cellular stress and mitochondrial impairment, particularly under metabolic and inflammatory conditions, and offers a comprehensive evaluation of mitochondrial disease potential [211]. Despite their use, these markers lack mitochondrial specificity due to confounding factors such as systemic inflammation. Integrating multi-omics technologies with traditional biomarkers may help overcome current limitations, enabling earlier and more precise diagnoses.

#### 4.1.2. mtDNA-CN as a Non-Invasive Diagnostic Tool

mtDNA-CN is indicative of mitochondrial biogenesis and function, serving as a potential biomarker for CVDs due to its role in energy metabolism and oxidative stress. mtDNA-CN, ranging from 2 to 10 copies per mitochondrion, serves as a surrogate marker for mitochondrial function. Its association with CVD has garnered significant interest in recent years. mtDNA-CN depletion may impair cardiomyocyte energetics and exacerbate the progression of CHD and adverse remodeling. Peripheral blood mtDNA-CNs have gained attention as a non-invasive diagnostic tool for CHD. Studies have demonstrated that reduced mtDNA-CN correlates with CHD severity and elevated cardiovascular risk [212] and is linked to peripheral arterial disease (PAD) and a two-fold increase in mortality risk [213]. Notably, mtDNA-CN undergoes dynamic changes with disease progression from stable angina to acute MI, suggesting its application in monitoring CHD progression [214]. Moreover, large-scale cohort studies support these associations [215,216,217]. mtDNA-CNs offer early detection potential (preceding structural changes) and cost-effective profiling using peripheral blood. However, methodological variability requires standardized protocols. mtDNA-CNs may outperform conventional biomarkers such as brain natriuretic peptide in detecting CVD at earlier stages [218]. Crucially, the clinical prognostic extrapolation of these findings necessitates large-scale multi-center prospective cohorts to establish causal inference between mtDNA-CN and cardiovascular pathogenesis. Such validation studies must systematically address three translational imperatives: (1) determination of pathologically significant mtDNA-CN thresholds; (2) assessment of population-specific predictive validity across ethnically diverse cohorts; (3) development of stratified risk algorithms capable of differentiating inter-patient risk gradients—prerequisites for achieving generalizable clinical implementation. For instance, the mtDNA-CN in circulating cell-free DNA and extracellular vesicles was higher in subjects with a history of CVD than in controls, suggesting a pathophysiological role in inflammation [219]. In veterans with post-traumatic stress disorder (PTSD), adjusted analyses revealed lower levels of circulating cell-free mtDNA (ccf-mtDNA), which correlated with increased glucocorticoid sensitivity [220].

#### 4.1.3. Mitochondrial Gene Mutations and CVD Risk

Mitochondrial gene mutations further underscore the genetic basis of CVD. Since 1981, seminal work has established mtDNA mutations as contributors to human pathology [221]. CVDs-related mtDNA mutations can be divided into numerous broad categories, each with a different mechanism of action: (1) predicted tRNA mutations disrupt base pairing at the affected site, potentially changing the secondary structure of the tRNA, leading to faster degradation and subsequent reduction in mitochondrial protein levels [222]; (2) mutations in the OXPHOS component reduce ATP synthesis and increase ROS production; (3) d-loop mutations interrupt the normal mtDNA replication process, leading to a reduction in mtDNA-CN [223]. Notably, most identified mtDNA mutations are either non-pathogenic or mild; therefore, they may not be able to develop a pathogenic phenotype [224]. Specific mutations, such as m.5725T>G in tRNA genes, disrupt mitochondrial function by altering the tRNA structure [189], whereas others, such as m.3243A>G and m.8839G>C, are associated with AS manifestations, including carotid stenosis and vascular dementia [225]. These mutations impair energy production, amplify oxidative stress, and dysregulate signaling pathways, thereby accelerating coronary AS. Carriers exhibit a 2–3-fold higher CHD risk and earlier disease onset (by 5–10 years), highlighting the potential of mtDNA mutation detection for genetic risk assessment and early intervention. For instance, the A3397G mutation in the ND1 gene has been identified in patients undergoing CABG [28]. Specific mtDNA heteroplasmies in monocytes from obese and CVD patients are correlated with cardiovascular risk factors [29]. The ALDH2 rs671 polymorphism has also been identified as a predictor for pulmonary hypertension of left heart disease (PH-LHD) [226]. Furthermore, transcriptome-wide association studies (TWAS) have identified susceptibility genes associated with erectile dysfunction, such as LCLAT1, which may be involved in cardiovascular health by affecting mitochondrial function and lipid metabolism [227].

### 4.2. Exploring Therapeutic Strategies Based on the Regulation of Mitochondrial Function

Mitochondrial dysfunction has emerged as an important therapeutic target in CVD, with current strategies focusing on key components, including ETC and mitochondrial dynamics. These elements play pivotal roles in the progression of CVD and are central to energy production and cellular homeostasis. Despite these efforts, no mitochondria-specific therapies have been approved for clinical CVD management [228]. However, advances in emerging technologies, such as mitochondrial replacement therapy, hold promise for future cardiovascular treatments [229].

#### 4.2.1. Mitochondria-Targeted Pharmacological Interventions in CVDs

**Figure 5:**
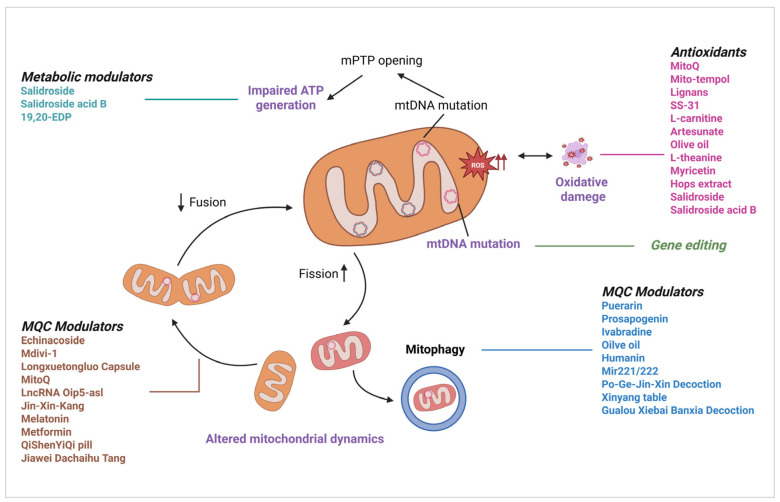
Mechanisms of mitochondrial dysfunction and current therapeutic strategies. A schematic overview illustrating the key mechanisms contributing to mitochondrial dysfunction, including impaired OXPHOS, ROS overproduction, mtDNA damage, and disrupted mitochondrial dynamics, as well as targeted therapeutic interventions such as antioxidants, metabolic modulators, and MQC enhancers. Abbreviations: ATP, adenosine triphosphate; mPTP, mitochondrial permeability transition pore; MitoQ, mitoquinone; mPTP, mitochondrial permeability transition pore; MQC, mitochondrial quality control.

Mitochondrial dysfunction has emerged as an important therapeutic target in CVDs, and several pharmacological agents have demonstrated potential to improve mitochondrial function and protect against myocardial injury through distinct mechanisms (Figure 5). We summarized the key mitochondria-targeted therapeutic drugs and their mechanisms of action in CVDs (Table 1).

A. Mitochondrial Antioxidants

Dysfunctional mitochondria produce significant amounts of ROS, which promote the development of CVD. Therefore, antioxidants are particularly important potential targets for the treatment of CVD. CoQ10, an important component of ETC, has demonstrated potential in preclinical and clinical studies by reducing oxidative stress [230], attenuating myocardial inflammation, and improving cardiac function in myocarditis patients [231]. However, larger trials are required to validate these results. The development of MitoQ, a novel mitochondria-targeted antioxidant, represents a significant advancement, with preclinical studies demonstrating its ability to (1) selectively accumulate in mitochondria, (2) scavenge ROS at their source [232], (3) reduce infarct size by 40–50% in I/R models [233], (4) improve cardiac function in pressure-overload HF [234], and (5) inhibit AS progression by protecting endothelial cells [235]. These findings have progressed to clinical trials [236]. For instance, MitoQ combined with alpha-lipoic acid improves oxidative stress and mitochondrial function in elderly rats with myocardial IRI [82]. Furthermore, the mitochondrial-targeted superoxide dismutase (SOD) mimetic mito-TEMPOL has been demonstrated to effectively attenuate nicotine-induced cardiac remodeling and dysfunction through selective scavenging of mtROS [237].

Lignans are flavonoid antioxidants that protect against H_2_O_2_-induced oxidative stress by modulating the ROS-mediated P38 MAPK/NF-κB pathway. Lignans are valuable candidates for AS treatment due to their antioxidant properties, which can enhance endothelial function [238].

The mitochondria-targeting SS peptide exhibits potent antioxidant bioactivity with established cardioprotective efficacy across preclinical and clinical investigation paradigms. Mechanistic studies demonstrate that its synthetic analog Bendavia (SS-31) functions as a selective mitochondrial respiratory modulator that suppresses electron transport chain uncoupling, attenuates ROS generation, and preserves oxidative phosphorylation integrity [239,240]. This multimodal action maintains mitochondrial bioenergetic fidelity and significantly reduces endothelial apoptosis under pro-oxidant conditions [241]. The SS peptide is an antioxidant active peptide that enters the mitochondria. Its cardiovascular efficacy has been widely reported in animal models and clinical trials. For instance, SS-31 inhibits mitochondrial respiratory chain uncoupling and ROS production in endothelial cells, protects ATP synthesis and mitochondrial function, and reduces EC apoptosis under oxidative stress [242]. Clinically, Elamipretide has been investigated in phase 3 trials for conditions including HFpEF and primary mitochondrial myopathy [243], though it has faced challenges in meeting primary endpoints, underscoring the complexities of targeting mitochondrial dysfunction in heterogeneous patient populations.

L-carnitine improves mitochondrial resilience by upregulating SOD activity and AMPK-dependent pathways [244]. Other natural products with antioxidant potential include: L-theanine [148], Puerarin [89], LTC [81], olive oil [37], hops extract [149], myricetin [150], Salidroside [45], Salvianolic acid B [151], shikonin [196], and GXBD acting via ALDH2 activation [195].

B. MQC Modulators

In addition to antioxidant therapy, another important therapeutic strategy involves increasing the number of mitochondria in the cell by promoting mitochondrial biogenesis. This approach reduces biological damage caused by mitochondrial damage or defects. PGC-1α has previously been identified as a significant regulator of mitochondrial biogenesis and is being explored as a potential clinical target for the treatment of CVD. Melatonin can restore mitochondrial biogenesis and reduce reperfusion injury through the AMPK/PGC-1α pathway [245] and has also been demonstrated to attenuate myocardial IRI by improving mitochondrial dynamics and mitophagy through the PINK1/Parkin signaling pathway [246]. Metformin protects cardiomyocytes from oxidative damage and delays endothelial cell senescence by modulating the AMPK signaling pathway, enhancing PGC-1α expression, and promoting mitochondrial biogenesis [164]. Indeed, studies have suggested that SGLT2 inhibitors may increase autophagy, which facilitates the removal of damaged mitochondria and enhances mitochondrial turnover [16]. Notably, the gut microbiota-derived metabolite urolithin A promotes cardiovascular health by inducing mitophagy through PINK1 stabilization [247]. Drugs that modulate mitochondrial dynamics, such as M1 (a fusion promoter) and Mdivi-1 (a fission inhibitor), exhibit cardioprotective effects in post-MI models [77]. Traditional Chinese medicine compounds targeting specific pathways also have the potential to regulate MQC. For instance, QSYQ inhibits fission [80], PGJXD activates PINK1/Parkin-mediated mitophagy [93], XYT regulates RIPK3/FUNDC1-mediated mitophagy [94], and Jiawei Dachaihu Tang modulates the SIRT1/PGC-1α/TFAM/LON pathway [69]. The lncRNA Oip5-as1 prevents excessive fission by regulating Drp1 phosphorylation [83]. JXK improves mitochondrial function by inhibiting the CaN/Drp1 pathway [84]. Although these approaches offer transformative potential by addressing the underlying causes of myocardial injury, challenges remain in clinical validation, drug delivery optimization, and personalized treatment strategies. The integration of these therapies may redefine CVD management and shift from symptomatic relief to metabolic correction.

**Table 1:** Summary of Mitochondria-Targeted Therapeutic Strategies in Cardiovascular Disease.

Therapeutic Agent	Category	Mechanism of Action	Cardiovascular Application	Ref.
MitoQ	Antioxidant	Accumulates in mitochondria; scavenges ROS at their source.	IRI, Atherosclerosis, HF	[82,232,233,234]
SS-31 (Elamipretide)	Antioxidant	Stabilizes inner mitochondrial membrane; inhibits respiratory chain uncoupling and ROS production.	IRI, Endothelial Dysfunction	[239,240,241,242]
Metformin	MQC Modulator	Activates AMPK/PGC-1α pathway, promoting mitochondrial biogenesis.	Cardiomyocyte protection, endothelial senescence	[164]
SGLT2 Inhibitors	Metabolic Modulator	Increases fatty acid oxidation and ketogenesis; may enhance autophagy and mitochondrial turnover.	HF (all phenotypes)	[16,186,187]
Mdivi-1	MQC Modulator	Inhibits the fission protein Drp1, reducing mitochondrial fragmentation.	Post-Myocardial Infarction remodeling	[77]
Artesunate/Jiawei Dachaihu Tang	MQC Modulator	Activates SIRT1, leading to deacetylation and activation of PGC-1α and other metabolic regulators.	Atherosclerosis, HF	[33,69]
Colchicine	Anti-inflammatory	Inhibits NLRP3 inflammasome assembly, which is linked to mitochondrial DAMPs.	Atherosclerosis (secondary prevention)	[122,123]

AMPK, adenosine 5′-monophosphate-activated protein kinase; DAMPs, damage-associated molecular patterns; Drp1, dynamin-related protein 1; HF, heart failure; IRI, ischemia–reperfusion injury; MitoQ, Mitoquinone; MQC, mitochondrial quality control; NLRP3, NOD-like receptor protein 3; PGC-1α, peroxisome proliferator-activated receptor-γ coactivator-1α; ROS, reactive oxygen species; SGLT2, Sodium-Glucose Cotransporter-2; SIRT1, Sirtuin 1.

#### 4.2.2. Gene Therapy for Mitochondrial Dysfunction in Coronary Heart Disease: Current Progress and Future Directions

A. Mitochondrial Gene Editing

The advent of advanced gene editing technologies, particularly CRISPR-Cas9 systems [248], has transformed therapeutic approaches for CHD by facilitating the precise correction of mitochondrial genetic defects. This innovation holds significant potential for treating CHD cases rooted in mtDNA mutations, offering the potential for functional restoration at the molecular level. CRISPR-Cas9 technology allows the targeted editing of pathogenic mtDNA mutations [249] and nuclear genes encoding respiratory chain components. For instance, potential gene therapy strategies could involve siRNA targeting endothelial S1PR 2 [32] or inhibiting calpain-2-mediated JPH2 cleavage [61].

B. Gene Therapy Strategies to Enhance Mitochondrial Function

In addition to direct DNA repair, gene therapy can enhance mitochondrial function by overexpressing SOD2 to increase antioxidant capacity [211] and activating PGC-1α to stimulate mitochondrial biogenesis and improve cardiomyocyte energy metabolism. These novel gene therapy-based strategies have revolutionized the treatment of CVDs and are anticipated to be translated from laboratory to clinic in the near future, bringing hope to most patients with CVDs for its eradication.

C. Challenges in Clinical Translation

Although these approaches represent a paradigm shift from symptom management to root-cause treatment, clinical translation faces challenges, including the development of cardiac-specific delivery systems and the verification of long-term safety. With technological advances, mitochondrial gene therapy may transition from preclinical promise to clinical reality within the next decade, potentially revolutionizing CVD treatment.

## 5. Conclusions and Future Perspectives

Mitochondrial dysfunction is a critical factor in CVD pathogenesis as it disrupts ATP production, amplifies oxidative stress, and initiates apoptotic pathways. These disturbances compromise cardiac cellular integrity, exacerbate ischemic injury, and accelerate myocardial remodeling. Recent advances in mitochondria-targeted therapies, including antioxidants, metabolic modulators, and gene-based interventions, offer promising avenues for improving clinical outcomes in CVDs. However, several challenges remain, including the limited therapeutic specificity, off-target effects, and translational barriers.

Future research must prioritize three key areas to bridge these gaps: (1) Mechanistic Elucidation: investigating the molecular interplay between mitochondrial dysfunction and CVD progression with a focus on mtDNA integrity, mitophagy regulation, and redox homeostasis. (2) Therapeutic Innovation: The development of precision therapies such as mitochondria-targeted antioxidants (for instance, MitoQ) and modulators of mitochondrial dynamics (such as Drp1 inhibitors) to restore bioenergetic stability. (3) Clinical Translation: Optimizing delivery systems, including nanoparticle carriers, and developing personalized strategies based on metabolic and genetic profiling.

Advancing these research priorities will be pivotal in transforming CVD management and transitioning from symptomatic relief to disease-modifying interventions, thereby improving patient survival and quality of life.

Despite the significant progress in elucidating the role of mitochondrial dysfunction in CVDs, key knowledge gaps persist, necessitating interdisciplinary efforts to drive translational breakthroughs. Three critical areas warrant immediate attention: (1) System Biology and Precision Medicine: It is imperative to conduct large-scale multi-omics studies that integrate whole-genome sequencing, epigenetics, and single-cell transcriptomics to understand how mitochondrial genetic variants interact with nuclear DNA and environmental factors to affect CVD susceptibility. (2) Next-Generation Diagnostics: Cutting-edge platforms combining nanotechnology-based mitochondrial metabolite sensors with artificial intelligence-powered imaging analytics promise to revolutionize CVD diagnosis and monitoring. Real-time, ultra-sensitive assessments of mitochondrial function at single-cell resolution could significantly enhance early disease detection and treatment stratification. (3) Therapeutic Frontiers: Future mitochondria-targeted therapies must surpass conventional pharmacology. For instance, the design of next-generation mitochondrial-targeted drugs to selectively modulate respiratory chain function, dynamics, and quality control mechanisms; leveraging CRISPR-derived gene editing for scarless correction of pathogenic mtDNA mutations, creating a foundation for molecular-level CVD therapies; and exploring mitochondrial transplantation and engineered stem cells to restore myocardial bioenergetics and function.

However, the path to its clinical translation remains complex. Optimizing mitochondria-targeted delivery systems, establishing standardized diagnostic protocols, and developing regulatory frameworks for novel therapeutics are significant challenges. Simultaneously, emerging opportunities lie in synergizing these approaches, such as integrating gene therapy with metabolic modulation or combining AI-powered diagnostics with personalized treatment strategies. Addressing these scientific frontiers holds the potential to transition CVD management from symptomatic control to precision mitochondrial medicine, thereby redefining treatment paradigms and improving long-term therapeutic outcomes.

## Data Availability

No new data were created or analyzed in this study.

## Abbreviations

The following abbreviations are used in this manuscript:

AHR	Aryl Hydrocarbon Receptor
ANT	Adenine Nucleotide Translocase
AKAP1	A-Kinase Anchoring Protein 1
ALDH2	Aldehyde Dehydrogenase 2
AMPK	Adenosine 5′-monophosphate-activated Protein Kinase
AS	Atherosclerosis
ATP	Adenosine Triphosphate
BBC3	BCL2 Binding Component 3 (also known as PUMA)
BDR	Benzodiazepine Receptor
BNIP3	BCL2 Interacting Protein 3
BNP	Brain Natriuretic Peptide
BYHWD	Buyang Huanwu Decoction
Ca^2+^	Calcium ions
CABG	Coronary Artery Bypass Grafting
CAD	Coronary Artery Disease
CaN	Calcineurin
CCND1	Cyclin D1
ccf-mtDNA	Circulating Cell-Free Mitochondrial DNA
cGAS	Cyclo-GMP-AMP Synthase
CHD	Coronary Heart Disease
CHF	Chronic Heart Failure
CMD	Coronary Microvascular Disease
CoQ10	Coenzyme Q10
CRISPR	Clustered Regularly Interspaced Short Palindromic Repeats
CUMS	Chronic Unpredictable Mild Stress
CVDs	Cardiovascular Diseases
CycD	Cyclophilin-D
DAMP	Damage-Associated Molecular Pattern
DNA	Deoxyribonucleic Acid
DNM2	Dynamin 2
DRP1	Dynamin-Related Protein 1
ECFCs	Endothelial Colony-Forming Cells
ECM	Extracellular Matrix
ED	Erectile Dysfunction
19,20-EDP	19,20-epoxydocosapentaenoic acid
eNAMPT	Extracellular Nicotinamide Phosphoribosyltransferase
ER	Endoplasmic Reticulum
ERRs	Estrogen-Related Receptors
ETC	Electron Transport Chain
FA	Fatty Acid
FAO	Fatty Acid β-oxidation
FGF-21	Fibroblast Growth Factor 21
Fis1	Fission Protein 1
FOXO3a	Forkhead Box O3a
FUNDC1	FUN14 Domain Containing 1
GAS6	Growth Arrest-Specific 6
GDF-15	Growth Differentiation Factor 15
GXBD	Gualou Xiebai Banxia Decoction
H_2_O_2_	Hydrogen Peroxide
HF	Heart Failure
HFmEF	HF with Mid-range Ejection Fraction
HFpEF	Heart Failure with Preserved Ejection Fraction
HFrEF	HF with Reduced Ejection Fraction
HO-1	Heme Oxygenase-1
ICAM-1	Intercellular Adhesion Molecule 1
IFN	Interferon
IL-1β	Interleukin-1 beta
IL-6	Interleukin-6
IL-10	Interleukin-10
IMM	Inner Mitochondrial Membrane
i-NOS	Inducible Nitric Oxide Synthase
I/R	Ischemia–Reperfusion
IRI	Ischemia–Reperfusion Injury
IVUS	Intravascular Ultrasound
JNK	Jun N-Terminal Kinase
JPH2	Junctophilin-2
JXK	Jin-Xin-Kang
KLF4	Krüppel-Like Factor 4
LCLAT1	Lysocardiolipin Acyltransferase 1
LDL	Low-Density Lipoprotein
LETM1	Leucine Zipper EF-Containing Transmembrane Protein 1
lncRNA	Long Non-Coding RNA
LPS	Lipopolysaccharide
LTC	Longxuetongluo Capsule
MAC	Mitochondrial Apoptosis Channel
MAP1LC3B	Microtubule Associated Protein 1 Light Chain 3 Beta
MAPK	Mitogen-Activated Protein Kinase
MARCHF5	Membrane Associated Ring-CH-Type Finger 5
MCU	Mitochondrial Calcium Uniporter
Mdivi-1	Mitochondrial Division Inhibitor 1
Mff	Mitochondrial Fission Factor
Mfn1/2	Mitofusins 1/2
MI	Myocardial Infarction
MIMP	Mitochondrial Inner Membrane Permeabilization
MitoQ	Mitoquinone
mito-TALENs	Mitochondrial-Targeted Transcription Activator-Like Effector Nucleases
MLKL	Mixed Lineage Kinase Domain-Like Protein
MMP	Mitochondrial Membrane Potential
MMPs	Matrix Metalloproteinases
MnSOD	Manganese Superoxide Dismutase
MOMP	Mitochondrial Outer Membrane Permeabilization
MPO	Myeloperoxidase
MPC	Mitochondrial Pyruvate Carrier
mPTP	Mitochondrial Permeability Transition Pore
MQC	Mitochondrial Quality Control
MRPL12	Mitochondrial Ribosomal Protein L7/L12
Mst1	Mammalian Ste20-Like Kinase 1
MTFP1	Mitochondrial Fission Process 1
mtDNA	Mitochondrial DNA
mtDNA-CN	Mitochondrial DNA Copy Number
mtROS	Mitochondrial Reactive Oxygen Species
Mzb1	Marginal Zone B And B1 Cell-specific Protein
ND1	NADH Dehydrogenase Subunit 1
NF-κB	Nuclear Factor-κ b
NHE	Na^+^/H^+^ Exchanger
NLRP3	NOD-Like Receptor Protein 3
NMN	Nicotinamide Mononucleotide
NO	Nitric Oxide
NOX2	NADPH Oxidase 2
NRF1/2	Nuclear Respiratory Factors 1/2
Nrf2	Nuclear Factor Erythroid 2-Related Factor 2
NSmase	Neutral Sphingomyelinase
Oip5-as1	Opa Interacting Protein 5 Antisense RNA 1
OMM	Outer Mitochondrial Membrane
OPA1	Optic Atrophy 1
ox-LDL	Oxidized Low-Density Lipoprotein
OXPHOS	Oxidative Phosphorylation
p38 MAPK	p38 Mitogen-Activated Protein Kinase
PAD	Peripheral Arterial Disease
PFOS	Perfluorooctane Sulfonate
PFOSA	Perfluorooctane Sulfonamide
PGAM5	Phosphoglycerate Mutase Family Member 5
PGC-1α	Peroxisome Proliferator-Activated Receptor-γ Coactivator-1α
PGJXD	Po-Ge-Jiu-Xin Decoction
PH-LHD	Pulmonary Hypertension due to Left Heart Disease
PHS	Pulmonary Hypertension Syndrome
PI3K	Phosphatidylinositol-3-Hydroxykinase
PINK1	PTEN-Induced Kinase 1
PKC	Protein Kinase Cepsilon
PM2.5	Particulate Matter 2.5
PPARα	Peroxisome Proliferator-Activated Receptor Alpha
PPARγ	Peroxisome Proliferator-Activated Receptor Gamma
PRRs	Pattern Recognition Receptors
PTSD	Post-Traumatic Stress Disorder
PUMA	p53 Upregulated Modulator of Apoptosis (also known as BBC3)
QSYQ	QiShenYiQi Pills/Dripping Pills
QTc	Corrected QT interval
Rap1	Ras-Related Protein 1
RHO/ROCK1/DRP1	Ras Homolog Family Member A/Rho-Associated Coiled-Coil Containing Protein Kinase 1/Dynamin-Related Protein 1 pathway
RIPK3	Receptor-Interacting Protein Kinase 3
ROS	Reactive Oxygen Species
RYR	Ryanodine Receptor
S1PR2	Sphingosine-1-Phosphate Receptor 2
sGC	Soluble Guanylate Cyclase
SGLT2	Sodium-Glucose Cotransporter-2
SIRT1/2/3/5	Sirtuin 1/2/3/5
siRNA	Small Interfering RNA
SMCs	Smooth Muscle Cells
SOCS1	Suppressor of Cytokine Signaling 1
SOD	Superoxide Dismutase
SOD2	Superoxide Dismutase 2
SR	Sarcoplasmic Reticulum
SS-31	Elamipretide (also known as Bendavia)
STING	Stimulator of Interferon Genes
TFAM	Mitochondrial Transcription Factor A
TLR4/9	Toll-Like Receptor 4/9
TNF-α	Tumor Necrosis Factor-alpha
tRNA	Transfer Ribonucleic Acid
TWAS	Transcriptome-Wide Association Studies
VCAM-1	Vascular Cell Adhesion Molecule 1
VDAC	Voltage-Dependent Anion Channel
VDAC1	Voltage-Dependent Anion Channel 1
VSMCs	Vascular Smooth Muscle Cells
XYT	Xinyang Tablet
